# Notes and News

**Published:** 1889-12-21

**Authors:** 


### Notes and News.

Collected and Communicated.

Madame Carnot is practical with her Christmas charities. She has invited 400 poor children to Paris from the provinces, and, besides the usual feasting and Christmas trees, will give each child a Saving's Bank book with an inscription of a 10 franc investment.

A correspondent writes to say that in our late article on "Unpleasant Truths about Food and Luxuries," the statement about the adulteration of tea was far too sweeping. So far as Indian tea is concerned, as it leaves the garden and as it is sold in Mincing-lane it is absolutely unadulterated. With regard to China tea, well, we all know that "the heathen Chinee is peculiar," and the tea estates in China are not held by Europeans as they are in India. Let us hope that, after all, our luxuries are not so deleterious as some stern moralists would have us believe.

Last week the Hornsey Local Board formally opened its extensive new sanitary depot in the High-street. The buildings consist of a public mortuary, with coroner's court, a waiting-room, two mortuary chambers, post-mortem rooms, laboratory, and caretaker's cottage. In another range of buildings, a steam laundry, disinfector, fumigator, and ambulance sheds, together with a range of stabling for twenty horses and a horsekeeper's cottage; finally, buildings for the treatment of house refuse—viz.: furnace-house, engine-house, cremator, mill-house, boiler-house, cart sheds, smithy, carpenters' workshop, stores, and a chimney shaft. The buildings were all open for inspection, and seemed to be fitted up in the most perfect manner, and so arranged as to effectually answer that troublesome question, "What are we to do with our refuse?"

The Lord Mayor presided over the annual meeting of the Hospital Sunday Fund at the Mansion House last week. In moving the adoption of the annual report of the council, Sir Sydney Waterlow congratulated the meeting on the success attained by the fund during the past year. Their income had steadily increased from year to year, and though the amount had not yet reached the sum which the great metropolis ought to contribute towards the support of hospitals and similar institutions, it was not for want of zeal on the part of those who preached on behalf of the fund. Speaking for the council, he would warmly thank those who made collections for the fund. Last year they collected about £1,100 more than in any previous year, though they had no legacies of importance. A large number of ministers of different denominations were present and several addressed the meeting, including Canon Fleming and Dr. Allon.

The Destitute Children's Dinner Society, of which the Baroness Burdett-Coutts is president, issues its twenty-third annual report. The work of the society has steadily increased, and 24,906 more dinners were given this year than last. While appealing for further support, the committee are most grateful for the grants of money given by different City Companies, and for the sum of £150 collected at the Stock Exchange.

Dublin Orthopœdic Hospital treated 121 intern patients last year. Of this number sixty-four were discharged cured, twenty were improved, nine incurable cases were relieved, and twenty-eight remained in the hospital when the year ended. It is noteworthy that a death has not occurred in this institution during the year; nor, indeed, for the last twenty months. The out-patient department is largely attended. The hospital is free from debt and wishes to keep so, therefore a ball in its aid will be given this winter.

On Saturday last a deputation of the Society for the Prevention of Hydrophobia and Reform of the Dog Laws, headed by Sir Henry Roscoe, and including Sir Spencer Wells, Dr. Farquharson, M.P., Prof. Pembertley, Dr. Sinclair Thompson, Prof. Horsley, Mr. H. Trueman Wood, F.S.A., Mr. Briton Riviere, R.A., and Mr. Frank Kerslake, waited upon Mr. Chaplin. The object of the deputation was to secure compulsory muzzling of dogs throughout the kingdom. Mr. Chaplin said the Government had the subject under consideration, and would shortly decide on some form of action.

At the late annual meeting of Coventry and Warwickshire Hospital, a good report was submitted showing that the hospital is in a healthy and sound position, both financially and otherwise. The financial year opened with a balance due to the treasurer of £120 8s. 2d., and closed with a debit balance of £123 11s. 1d. The cost of provisions, which in the previous year amounted to £1,114 9s. 2d., had during the past year amounted to £1,123 17s. 10d. The chairman, Mr. Dugdale, Q.C., M.P., made a bright and clever speech, and Dr. Milner Moore returned thanks for the medical staff. The generosity of Mrs. Gulson was highly commended.

The opening ceremony of King's Cross Hospital, Dundee, was performed by Lord Provost Hunter on December 11. The visitors were conducted over the buildings by Mr. Mackison, architect; Mr. Thomson, his assistant; and Dr. Anderson, medical officer of health. The utmost satisfaction was expressed by everyone, especially with the reception wards and the pavilions, which are large, airy, and well ventilated. After the inspection the company partook of luncheon, provided by ex-Bailie Ogilvie, and then the speeches began. Everybody congratulated and praised everybody all round, and Dr. Anderson actually hoped that the hospital would enlarge and prosper. That would suit the doctor, doubtless, but not the ratepayers nor the patients.

The annual report of Ayr County Hospital is out. During the year 484 cases have been treated in the wards of the hospital—one less than last year, but an increase of 48 cases as compared with the year 1887. Including the dispensary patients, the total number of cases for the year amounted to 1,683. The total ordinary and special income for the year amounts to £1,528 4s. 9½d. The total ordinary and special expenditure amounts to £1,759 19s. 0½d.; adding to this the amount (£13 19s. 11d.) due to the bank on maintenance account at the close of last year, there is a deficiency of income to meet the expenditure to the extent of £245 14s. 2d. There is still a heavy debt on the building fund of the new hospital, but the county gentlemen are coming forward generously to reduce it.

The following vacancies are announced this week, the amount of the salary in each case being given after the name of the institution:

**Resident Medical Officers.**

Derbyshire General Infirmary. Assistant House Surgeon—board, &c.
West London Hospital. House Surgeon and House Physician—board, &c.
Monsall Fever Hospital. Assistant Medical Officer—£100, board, &c.
Leeds General Infirmary. Ophthalmic Officer—£50, board, &c.
Liverpool Northern Hospital. House Surgeon—£100, board, &c.
Liverpool Northern Hospital. Assistant House Surgeon—£70, board, &c.
Birmingham General Hospital. Two Assistant House Surgeons—board, &c.
Victoria Hospital for Children, Chelsea. House Physician—£50, board, &c.
Victoria Hospital for Children, Chelsea. House Surgeon—£50, board, &c.
Northampton General Infirmary. House Surgeon—£125, board, &c.
Northampton General Infirmary. Assistant House Surgeon—£80, board, &c.
East London Hospital for Children. House Surgeon—board, &c.
Burnley Victoria Hospital. Medical Officer—£80, board, &c.
Royal Westminster Ophthalmic Hospital. Clinical Assistants.
Cheltenham General Hospital. House Surgeon—£80, board, &c.

**Non-Resident Medical Officers.**

Chelsea Hospital for Women. Assistant Physician.
Great Northern Central Hospital. Physician to Out-patients.
Mercer's Hospital, Dublin. Physician.
University of Edinburgh. Examiner in Clinical Surgery—£75.
Hospital for Sick Children, Great Ormond-street. Surgical Registrar—£40.
Chelmsford Union. Medical Officer—£140.

The tenth annual exhibition of home-made and other toys for distribution amongst the children in the various London hospitals, workhouses, workhouse schools, and infirmaries will be held in the Grosvenor Gallery, Bond-street, on Monday and Tuesday next. *Truth* hopes to distribute 22,000 toys to the children personally, and another 1,000 of the more handsome toys as permanent gifts to the various hospitals and infirmaries. The same anonymous donor has again given 10,000 new sixpences to be distributed.

We are all very charitably inclined just now, so the Mendicity Society, of 8, Fisher-street, Red Lion-square, is sending forth a reminder that the charity which breeds destitution is mere selfishness. To give to beggars indiscriminately is as foolish at Christmas as at other times, and those ladies who have not the heart to refuse the wretched-looking creatures who infest the streets should provide themselves with the food tickets issued by this society, which at least prevents the alms being spent in drink.

We are indebted to the *Times* for the following items of Indian news. A meeting of the Bombay branch of the Dufferin Fund was held in Bombay, Dec. 11. Large donations were promised, amongst others one of £30,000, and another £10,000. A full dress parade of the troops was held at Poonah on the 6th inst., to witness the presentation, by the Duke of Connaught, of the Victoria Cross to Surgeon Crimmin. The Duke complimented him on the conspicuous bravery he had displayed in risking his life to attend the wounded under heavy fire.—The Chloroform Commission has closed. Experiments upon 610 animals have conclusively demonstrated that there is no danger to the heart direct in applying chloroform. The Nizam has been present and has taken great interest in the experiments.

The House of Shelter, Waterloo-street, Commercial-road, E., acts in concert with other charities: thus the Charity Organisation Society, last year, sent various cases to the shelter while they made further investigations. They also investigated the character of some of the applicants to the shelter. Working in this way much good is done, and now that the cold weather is upon us funds are badly needed, also old clothes, especially old boots.

The Providence Hospital bazaar, at St. Helens, was a great success, and on the first day only over £200 was cleared towards the Free Hospital. The hospital was founded by and is nursed by a Catholic sisterhood, but is undenominational in its work. The bazaar was open four days, and beside the pretty flower, refreshment, and other stalls, there were frequent concerts and entertainments. The whole reflected great credit on the secretary, Mr. Aloysius Williams.

The cause of the new infirmary for Stafford is being warmly taken up, and subscriptions are fast coming in, the Earl of Dartmouth giving £100. If £5,000 is subscribed the governors of the infirmary will have ample funds for the erection of a new and modern hospital upon a better and healthier site; as that sum, coupled with what the old building and site may be expected to realise, will suffice. The Society of Amalgamated Engineers in Stafford has taken a great interest in the infirmary, and has promised to assist in raising the funds now required, and the committee trust that this excellent example may be followed by other trades.

Deptford Medical Mission is appealing for £500 before December 31 to close its first half year free of debt. The mission works in a very poor part of London, and has the hearty support of the medical men of the neighbourhood. The mission-room is at 188, High-street, Deptford. Great ignorance seems to exist in the neighbourhood, for the medical officer reports the following home prescriptions: "One day I had a case in which a child had been treated for eczema as follows: Cabbage-leaves baked before a clear fire, and applied as hot as possible to the affected parts. It is needless to say that this very painful treatment was productive of much harm. Also, 'Paraffin well rubbed into the chest and back' for bronchitis was anything but a sweet and happy thought. 'Gin neat,' for indigestion, was far from useful, but here the wish may have been father to the thought."

The Leprosy (Father Damien) Fund, of which the Prince of Wales is president, propose: 1. That a sum of not less than £500 be appropriated to a memorial to be erected to Father Damien in some public place, and that estimates and designs for a statue in stone, marble, or bronze be invited from Mr. Gilbert. 2. (*a*) That a fund be formed, the interest of which shall be devoted to the medical treatment and care of indigent British lepers in the United Kingdom. (*b*) And that a sum of money be set apart and placed under the control of trustees for the endowment of two studentships, one student to make the United Kingdom and the remainder of Europe his field of investigation, and the other to go abroad and study the disease in China, the colonies, and elsewhere. The studentships to be held for a period of three years, to be renewed by the trustees if thought desirable. 3. That a commission be appointed, for not less than one year, consisting of three members, one to be named by the Royal College of Physicians, one by the Royal College of Surgeons, and one by the General Committee of the Leprosy (Father Damien) Fund, to go out to India for the purpose of investigating the disease of leprosy there, and that the Indian Auxiliary Committee be requested to add two members to this commission. Donations amounting to nearly £4,000 have already been received.

